# Dr. Taft’s Address before the Mississppi Valley Ass’n of Dental Surgeons

**Published:** 1852-10

**Authors:** 


Dr. Taft's *Address before the Mississippi Valley Association of Dental Surgeons; Held Sept.* 8 *and* 9, 1852.

*Mr. President and Gentlemen:* At a former occasion it was my privilege to present for your consideration a few thoughts upon dentition; those thoughts were confined to one physiological point, viz: the eruption of the teeth. There are many other points which it would be interesting and in structive to examine minutely; this, however, would greatly transcend the design of this paper. We will consider one or two other physiological points, and then make a few remarks upon some pathological conditions that arise during dentition.

In the development of the teeth, though there is a comparative uniformity in many points, yet there is an infinite variety in others.

There is a comparative uniformity in the manner in which the teeth grow, in the condition of the primary rudiments, also in the order in which the respective parts are developed, the method of eruption, &c. In other respects there are endless variations, examples of which we find in the rapidity of growth, from the purative germ to the completion of the operation; the rapidity of growth at one period, and tardiness of that growth at another period, in the same subject. These variations result from the variety of circumstances in which various individuals are situated; together with an absence of uniform constitutional development and tendencies.

There are many circumstances that influence the development of the dental organism. The primary constitution of the child will determine to some extent the character of the teeth. If the constitution is good, and its chemical functions perfectly performed, we anticipate happy results in the general development. A constitution may be good, and yet the functional operations be perverted; the general organization may be apparently perfect, and yet a slight, imperceptible derangement or deficiency in some function, may occasion an ultimate result which will be very apparent.

The organism whose office it is to secrete the elements of denture, especially the earthy elements, may elaborate one or more of them in quantity too great, or too small, or may produce them in improper relative proportions to constitute a perfect organ. The elements may not only be relatively in excess, or deficient in quantity, but may also be of a deteriorated quality; the latter is probably much less frequent than the former: where either of these conditions is present, a perfect dental organism is impossible.

The animal matter that enters into the composition of dentine, is more easily deteriorated in quality than the earthy elements; when this is the case, the teeth will be more easily affected by decay.

The animal portion is the medium that sustains the vital organization of the teeth; the more perfect that medium, the stronger their vitality, and the better will they withstand the influence of external destructive agents.

A preponderance of gelatine gives a delicate transparent appearance, which is beautiful, but rarely remains so for any great length of time. Such teeth are commonly found in persons of a cachectic diathesis, and especially by those possessing a strong hereditary tendency to phthisis pulmonalis.

When there is a preponderance of the earthy bases, the teeth have a white and chalky appearance, and always yield rapidly to the ravages of decay.

Nature primarily established the proper relative proportions of elements, and a very slight deviation from that proportion would produce quite a different material; hence it is that different analysis made upon different teeth would not always give the same result.

If the animal elements exist in deteriorated quality, or in improper quantity, then the vital organization will be deficient, weak, and easily destroyed; they neither defend themselves, nor shield the other elements so well, as when in perfection, and hence the necessity of pursuing such a course as will establish the most perfect development.

The animal element is first supplied in the order of arrangement, and is ramified with nerves and vessels, the organs of vitality; and secondarily in order, is the deposition of the calcareous portion. In the formation of bone, the arrangement of the gelatinous portion is first in order, and then the earthy materials are supplied. The production of dentine is not an exception to this rule.

The function of these two component parts, is to sustain each other; the animal portion sustains the vital organization, the calcareous part gives strength, beauty, and utility to the organ.

Dentition is one of the most critical periods of life to which nature's operations subjects the human being; it is not, however, *per se*, necessarily so. The difficulties that are exhibited during dentition, are not the direct results of that process.

This period is one in which the energies of the system appear to be largely drawn upon; and nature's demands and requirements should be complied with to the full, and even this would not be sufficient to avert the evil at all times; for there may be, and often is in the system hereditary tendencies to diseased action, that requires all the vital powers of the system to counteract; which, when drawn to some other point, these tendencies manifest themselves in actual disease.

When these exist, then in addition to fulfilling the requirements of nature, in regard to food, drink, clothing, sleep, exercise, and pure air, such means should be resorted to as would most effectually prevent the development of any predisposition in diseased action; and also to sustain and strengthen the vital power.

During the period of dentition, diseased action may be induced by disregarding nature's requirements, independent of any hereditary predisposition, or when it did not exist. Improper nourishment, either in quality or quantity will produce disease in the system, when all the vital powers are concentrated upon its sustentation, much more than when these are drawn from this and fixed upon the accomplishment of some important process.

It would be impossible in a paper of this design, to descend to all the minutuæ, and if it were, it would be unavailing, for duty is much better known than practiced; yet a few remarks upon *some* of those things that may avert or control those evils that arise, or are liable to arise during dentition, may not be out of place.

During the earlier periods of dentition, nature supplies the child with the appropriate nourishment, particularly so if the course of the mother or nurse has been one of care and discrimination.

The practice of giving artificial aliments in the early periods of infancy, or before there is a desire manifested by the child for it, is one of exceedingly doubtful propriety; this opinion is strongly supported by the fact, that almost all infants sustained by artificial aliment have difficult dentition.

It is when and after the artificial aliment is substituted for the simple nourishment which sustained the earlier existence, that the difficulty in this respect arises. All the circumstances that surround the case, admonish to simplify the diet at this time as much as possible, especially in regard to variety; constitutional peculiarities should not be disregarded in the selection of food; that food which would be altogether proper for a child of one temperament, would be entirely unsuited for one of an opposite, or different temperament.

Sleep, the great restorer, should be solicited at proper intervals, in pure air; and at such times as would tend most to repair the wasted energies of the system.

In regard to clothing, it should be so adjusted that all parts would be perfectly free from compression. So far as the temperature of the body is governed by clothing, it should be uniform; change of clothing should not be permitted more often than is necessary to fulfil the requirements of perfect cleanliness.

The frequent substitution of one kind of clothing for another is deleterious, and especially so when it disturbs the uniform temperature.

It is frequently the case, and particularly in cities, that the whole attire of infants is changed three or four times each day; this, with many other pernicious practices, produce a vastly greater amount of mortality in the ranks of infancy and childhood in cities than in the country.

